# The training and development needs of nurses in Indonesia: paper 3 of 3

**DOI:** 10.1186/1478-4491-4-10

**Published:** 2006-04-23

**Authors:** Deborah Hennessy, Carolyn Hicks, Aflah Hilan, Yoanna Kawonal

**Affiliations:** 1 School of Health Sciences, University of Birmingham, Birmingham, UK; 2 Independent consultant, Jakarta, Indonesia; 3 Officer, Indonesian Nurses' Association, Jakarta, Indonesia

## Abstract

**Background:**

Indonesia's recent economic and political history has left a legacy of widespread poverty and serious health problems, and has contributed to marked inequalities in health care. One means of responding to these challenges has been through a reconsideration of the professional roles of nurses, to enable them to deal with the range and complexity of health problems. However, there are currently a number of obstacles to achieving these aims: there is a serious shortfall in trained nurses; the majority of nurses have only limited education and preparation for the role; and there is no central registration of nurses, which means that it is impossible to regulate either the profession or the standards of care. This study aimed to establish the occupational profiles of each grade of nurse, identify their training and development needs and ascertain whether any differences existed between nurses working in different regions or within hospital or community settings.

**Methods:**

An established and psychometrically valid questionnaire was administered to 524 nurses, covering three grades and coming from five provinces.

**Results:**

Significant differences in job profile were found in nurses from different provinces, suggesting that the nature of the role is determined to some degree by the geographical location of practice. The roles of hospital and community nurses, and the different grades of nurse, were fairly similar. All nurses reported significant training needs for all 40 tasks, although these did not vary greatly between grade of nurse. The training needs of nurses from each of the provinces were quite distinct, while those of hospital nurses were greater than those of community nurses.

**Conclusion:**

The results suggest that the role of the nurse is not as diverse as might be expected, given the different levels of preparation and training and the diversity of their work environments. This may reflect the lack of a central registration system and quality framework, which would normally regulate clinical activities according to qualifications. The differences in training needs between subsections of the sample highlight the importance of identifying skills deficits and using this information to develop customized post-registration education programmes. Together, these results provide a rigorous and reliable approach to defining the occupational roles and continuing education needs of Indonesian nurses.

## Background

### Context

A range of geopolitical factors have affected the economy in Indonesia. Current poverty estimates suggest that almost a third of Indonesia's population are living below the World Bank poverty line [1], with all the implications this has for health status. Poverty levels have contributed to a number of serious health problems such as: sharp rises in communicable diseases, including TB, cholera and malaria [2,3] and acute respiratory illness [4]; compromised nutrition leading to Vitamin A and iron deficiencies, goitre and xerophthalmia [5]; and diabetes [6]. Not only do these problems pose serious risks for the population's health and the country' economic viability, they also present a significant challenge for the health service.

The situation is further compounded by the financial and organizational structure of the health care system, which includes the devolved responsibility to the provinces for raising a third of the income needed to manage local health issues. Limited financial resources, coupled with the teething problems of decentralization and a geographically diverse country, have meant that uptake of health care has also diminished by 25% since the economic crisis [7]. One consequence of these combined factors is inequitable provision in health care quality and accessibility, which in turn have contributed to the declining health status of the population. This contextual backcloth represents a huge challenge to the Indonesian government in terms of redressing inequalities and generally enhancing the standards of clinical provision.

### Health care in Indonesia

At an operational level, it also means that health care professionals have significant responsibility for managing the delivery of health care within a framework of limited provision and great environmental diversity. The health care system, widely accepted to be underfunded, with just 2% of the GNP being spent on health care, has created an obvious shortfall in equipment, supplies and health care personnel; moreover, it has affected their education and training.

Given that much of Indonesia's health care is delivered through an extensive primary care system which relies heavily on midwives and nurses for its efficacy and success, it seems self-evident to note that the preparation of these personnel is crucial to the effective functioning and delivery of clinical services. Moreover, because of the pressure on services and the wide variations in terrain and population levels, continual updating of health care professionals is required if they are to meet very specific local needs. Consequently, nurses must not only develop the clinical skills to manage serious diseases, but also must be sensitive to the cultural needs of the patient and family, while simultaneously operating in adverse environmental conditions with limited equipment and resources.

The challenge to both the government and the individual nurse is very considerable and means that not only is it essential that the basic training of nurses produce the essential competences required, but that an informed range of postbasic training or continuing professional development (CPD) is also required.

### Nurse training and service delivery in Indonesia

While much of the background information regarding the current position with regard to nurse training and service delivery have already been provided in paper 1 [8], it is perhaps worth reiterating a number of key points that have direct relevance to this paper.

• There is a serious shortfall (by international standards) of qualified nurses in Indonesia, with an estimated 50 nurses per 100 000 of the population.

• The majority of nurses (60%) are educated to high school level only, 39% have a diploma and 1% are graduates; these latter two groups typically move into education soon after completing their training. This means that not only do nurse educators have little clinical experience in the field, which could compromise the applicability of their teaching, but also that the majority of direct clinical care is delivered by the least qualified nurses. There appears to be little differentiation between clinical roles for the different levels of education.

• No central registration of nurses exists, which means it is impossible to regulate the profession, to enforce quality standards and to ensure accord between level of training and clinical activities undertaken.

• Like midwives [9], many nurses do not have a formal job description, which means that professional and competence boundaries may be exceeded, especially where there is pressure on services and limited resources. It also limits the effectiveness of the individual performance assessment (IPA) system (see below).

• Like all government employees, nurses are required to have an annual IPA as a mechanism by which quality standards can be monitored and maintained through CPD. The structure and framework of these IPAs varies greatly, which may affect nurses' understanding of their skill levels and development needs.

### The way forward

Together these factors mean that for nurse education and development to be optimized, it is essential that some index of current occupational profiles is obtained. This would capture the differences in role, dependent on locality of operation (urban/rural, hospital/community, etc.), which would enable the customization of basic and postbasic education provision to meet local needs; it would also afford the development of a structured job description and a benchmark against which to conduct IPAs. Furthermore, if training needs could also be collected, then this would enable the more systematic provision of CPD, through targeted interventions on reported skill deficits; this would also facilitate a more responsive approach to local needs.

### Aims of the current study

Consequently, the current survey was undertaken with 524 nurses from a number of provinces in Indonesia. The specific aims were:

• to obtain an occupational profile of nurses, by grade and work situation (hospital/community and province); this would serve to inform basic nurse training, subsequent job descriptions, and act as a framework for IPAs;

• to identify the training needs of nurses by grade and work situation (hospital/community and province), in order to establish where the key skill deficits lay and thence to develop continuing professional development packages, customized to local needs if necessary;

• to ascertain whether having a job description affected how nurses perceived their roles and training needs;

• to ascertain whether having an IPA affected how nurses perceived their current performance levels and future training needs.

## Methods

### Sample

Five hundred and twenty-four (524) nurses participated in the study. Of these, 115 were male and 409 female; they represented both hospital and community locations (n = 300 and N = 224, respectively) and five provinces (North Sulawesi n = 139, East Kalimantan n = 155, North Sumatra n = 190, West Java n = 15 and Djakarta Kota Indonesia (DKI) n = 25. Of the 524 nurses, 287 were at SPK level (High School Certificate in Health Care – three years post-junior high school), 197 at D3 level (three-year post-high school diploma) and 40 at S1 level (three to five-year post-high school, university degree in nursing). The samples from West Java and DKI comprised S1 nurses only.

As with the midwifery sample [9], the provinces were selected to cover key strata, such as urban/rural, developed/underdeveloped, dense/sparse population and terrain. The details are as follows:

• West Java: urban, developed, densely populated

• DKI: urban, developed, densely populated

• North Sumatra: urban, developed, densely populated

• East Kalimantan: rural, underdeveloped, sparsely populated

• North Sulawesi: rural, developed, sparsely populated.

### Materials

A self-report questionnaire was administered, which has been described in paper 1 [8]. The instrument comprises two main parts: a biographical section (to enable subsample comparisons) and a 40-item questionnaire. This instrument was modified from an established training needs analysis questionnaire [10] and covers core nursing tasks, which the respondents are required to rate in each of two ways: how important the task is to their work and how well they are currently performing it. Each rating is along a seven-point scale. The importance rating provides a job profile or role as perceived by the respondents and a comparison of the two ratings awarded to each task provides an index of training need, in that important tasks which are poorly performed indicate a significant training need [11].

### Procedure

The nurses were transported by bus, sea or river boat to a centre in each district, where a team of trained data collectors supervised the completion of the questionnaires. The data were entered into an SPSS database for analysis.

## Results

### The occupational profile of nurses

#### Whole sample

The importance ratings given by the whole sample to each of the items suggested that 34 tasks were considered to be extremely important, with ratings in excess of 6.0 (maximum = 7.0); the remaining six tasks were rated as important (range 5.5 – 5.9) and comprised: assessing costs and outcomes of procedures; undertaking administrative duties; making appropriate patient referrals; critically evaluating research; undertaking budget planning activities; planning patients' discharge. These results suggest that all tasks are considered to be either important or very important to the nurses' role.

To see whether the tasks fell into clusters of skills that could form the modules for a core nursing curriculum, the data were subjected to exploratory factor analysis, following a recommended protocol [12] and a Varimax rotation. With a KMO test of sampling adequacy of 0.951, Bartlett's test of sphericity p = 0.000 and eigen values in excess of 1.0, three factors emerged, which together accounted for 43.1% of the variance. The factors, with proposed names, are presented in Table 1, with factor loadings in brackets. These factors fall into three coherent clusters of skills that could inform both the core curriculum for basis nurse training and a outline framework of a job description.

**Table 1:** Factor structure of critical nursing competences

**Factor 1 (34.1% of the variance, Cronbach's α = 0.9090): Clinical care and service management**

Undertaking technical nursing procedures (0.655)
Assessing patients' psychological and social needs (0.645)
Recognizing and managing risk in clinical care (0.576)
Developing a shared mission of clinical goals (0.568)
Assessing patients' physical needs (0.552)
Showing patients and their families how to do things (0.536)
Establishing a relationship with patients (0.515)
Undertaking health promotion and prevention activities (0.512)
Analysing patient data (0.509)
Consulting with colleagues about care options (0.501)
Assisting patients in making informed choices (0.496)
Prioritizing work according to patients' needs (0.488)
Writing clinical, shift and other reports (0.480)
Working as a member of a team (0.477)
Using technical equipment (0.442)
Getting on with colleagues (0.441)
Making decisions about patients' clinical problems (0.437)
Designing systems for patient monitoring/observation (0.427)
Appraising own and others' performance (0.426)

**Factor 2: (5.1% of the variance, Cronbach's α = 0.8942): Managing change and the application of knowledge**

Actively assisting in change management activities (0.690)
Introducing new ideas into own clinical work (0.683)
Interpreting results from clinical investigations (0.657)
Inputting data into written or computerized records (0.620)
Instructing/training students/junior staff (0.576)
Undertaking budget planning activities (0.552)
Collecting own clinical/patient/surveillance data (0.547)
Planning patients' discharge (0.544)
Undertaking clinical examinations of patients (0.538)
Interpreting own patient data (0.525)
Developing joint working relationships with others (0.514)
Critically evaluating published research (0.501)
Locating and accessing equipment for clinical work (0.439)

**Factor 3 (3.5% of the variance, Cronbach's α = 0.7970): Case management and networking**

Applying pharmacology to practice (0.674)
Assessing costs and outcomes of procedures (0.631)
Undertaking administrative duties (0.627)
Liaising with other health care professionals (0.590)
Identifying areas worthy of investigation in practice (0.584)
Requesting laboratory investigations and results (0.549)
Making appropriate patient referrals (0.425)
Planning/organizing patients' treatment (0.388)

### Occupational profile of nurses: subsample comparisons

#### By province

The five provinces were compared on importance ratings for each of the 40 items, to establish whether there were any differences between the occupational roles of the nurses within these localities. A series of unrelated one-way ANOVAS was performed, with post hoc Tukey tests where significant results were obtained. Thirteen significant differences were found, which can be found in Table 2 (df = 4 and 519 in each case).

**Table 2:** Comparison of job profiles by province

**Item**	**F**	**P**	**Implication**
Assessing costs and outcomes of procedures	5.294	0.000	Significantly more important in 3 than in 2 and 5
Undertaking administrative duties	8.306	0.000	Significantly more important in 2, than in 1,3 and 5; significantly more important in 1 and 3, than in 5
Consulting with colleagues about care options	2.493	0.042	No province significantly different from any other
Critically evaluating published research	3.400	0.009	Significantly more important in 2 than in 1
Planning/organizing patients' treatment	3.057	0.017	Significantly more important in 1,2 and 3 than in 5
Working as a member of a team	3.619	0.006	Significantly more important in 1 than in 3
Undertaking budget planning activities	7.044	0.000	Significantly more important in 2 than in 1 and 5; significantly more important in 1 and 3 than in 5;
Developing joint working relationships with others	6.869	0.000	Significantly more important in 2 than in 1; significantly more important in 1 than in 3
Introducing new ideas into own clinical work	4.511	0.001	Significantly more important in 2 and 3 than in 1
Actively assisting in change management activities	2.817	0.025	No province significantly different from any other
Interpreting own patient data	2.738	0.028	Significantly more important in 5 than In 1
Inputting data into written or computerized records	2.905	0.021	Significantly more important in 2 than in 1
Planning patients' discharge	3.516	0.008	Significantly more important in 5 than In 1

1 = East Kalimantan; 2 = North Sulawesi; 3 = North Sumatra; 4 = West Java; 5 = DKI

As these results derive from only five of the 34 provinces, they can be taken to indicate only that sufficient significant differences exist at regional level to consider customizing the core training to meet local needs and to modify the job descriptions with these variations in mind. As the samples from West Java and DKI were solely S1 nurses, the findings would suggest that these nurses have a better preparation for case management, interpreting patient data and planning patient discharge, but for this they would also need a better understanding of cost-effectiveness and administration, which is indicated in the findings in Table 2.

#### By work location (hospital versus community)

A series of unrelated t-tests was undertaken to compare the importance ratings given to each task, in order to provide an overview of the occupational roles of hospital and community-based nurses. Eight significant differences emerged, representing an 80% overlap in job profiles between these two groups. The differences are presented in Table 3 (df = 522 in each case).

**Table 3:** Comparison of job profiles of hospital and community nurses

**Item**	**T**	**P**	**Mean score: hospital**	**Mean score: community**	**Implication**
Undertaking administrative duties	3.874	0.000	5.33	5.92	More important for community nurses
Assessing patients' physical needs	-2.270	0.024	6.56	6.31	More important for hospital nurses
Making appropriate patient referrals	2.475	0.014	5.76	6.12	More important for community nurses
Recognizing and managing risk in clinical care	-2.065	0.040	6.47	6.24	More important for hospital nurses
Assessing patients' psychological and social needs	-2.656	0.008	6.49	6.20	More important for hospital nurses
Undertaking health promotion activities	2.430	0.015	6.28	6.52	More important for community nurses
Undertaking budget planning activities	2.206	0.028	5.48	5.83	More important for community nurses
Inputting data into written or computerized records	2.758	0.006	5.86	6.23	More important for community nurses

These results suggest that while 80% of the hospital and community nurses' roles are very similar, there are some areas of difference that could be used to tailor basic training and job descriptions.

#### By grade of nurse – SPK, D3 and S1

To identify whether the occupational roles of the three grades of nurse differed significantly, comparisons were made of the importance ratings awarded by each group, using a series of 1-way ANOVAS for unrelated designs, and post hoc Tukey tests where there were significant ANOVA results. Seven differences emerged which are presented in Table 4 (df values 2 and 521 in each case).

**Table 4:** Comparisons of job profiles by nursing grade

**Item**	**F**	**p**	**Implication**
Undertaking administrative procedures	5.807	0.003	Significantly more important for SPK nurses than for S1
Assessing patients' physical needs	5.864	0.003	Significantly more important for S1, followed by D3 and finally SPK
Consulting with colleagues	3.306	0.037	Significantly more important for SPK nurses than for S1
Planning/organizing patients' discharge	8.660	0.000	Significantly more important for SPK, followed by D3 and finally S1
Working as a member of a team	3.275	0.039	No significant differences between the groups
Undertaking budget planning activities	5.735	0.003	Significantly more important for SPK nurses than for S1
Interpreting own patient data	4.916	0.008	Significantly more important for S1 nurses than for SPK

These results suggest that there is significant commonality between the perceived roles of each grade of nurse; however, the differences in seven of the 40 tasks indicate that the different levels of training and educational preparation are reflected in how the roles are currently discharged. They might also support different job specifications for each grade.

### Training needs

#### Whole sample

To capture the training needs of the nursing sample, the performance ratings were compared with the importance ratings for each task [11], using a series of related t-tests. All 40 tasks were found to have a significant training need (p < 0.000 in each case), with the importance ratings for 34 of the items being in excess of 6.00, while for the remaining six, the importance scores ranged between 5.5 and 5.9 (see above). These training needs were then factor-analysed according to recommended protocol [12], using a Varimax rotation, to establish whether coherent clusters of training requirement existed that could inform continuing professional development (CPD).

Three factors emerged that accounted for 37.2% of the variance (KMO test of sampling adequacy = 0.942, Bartlett's test of sphericity = 0.000; eigen values >1.0). These are shown in Table 5, together with proposed names, Cronbach's α scores and factor loadings in brackets. These factors provide evidence of a coherent set of skill deficits that could be used to inform CPD modules.

**Table 5:** Factor structure of training needs

**Factor 1 (28.6% of the variance; Cronbach's α = 0.9265): Nursing care and leadership**

Interpreting patient data (0.683)
Developing joint working relationships (0.619)
Undertaking clinical examination of patients (0.619)
Making decisions about patients' clinical problems (0.618)
Collecting own clinical data (0.611)
Using technical equipment (0.604)
Assessing patients' psychological and social needs (0.587)
Undertaking technical nursing procedures (0.584)
Prioritizing work according to patients' needs (0.583)
Interpreting results from clinical investigations (0.583)
Showing patients and their families how to do things (0.576)
Actively assisting in change management activities (0.570)
Developing a shared mission of clinical goals (0.565)
Locating and accessing relevant equipment (0.564)
Writing clinical/shift/other reports (0.563)
Instructing/training students/junior staff (0.562)
Introducing new ideas at work (0.545)
Undertaking budget planning activities (0.532)
Inputting data into written or computerized records (0.531)
Undertaking health promotion/prevention activities (0.521)
Getting on with colleagues (0.490)
Working as a member of a team (0.486)
Planning patients' discharge (0.465)
Recognizing and managing risk in clinical care (0.450)
Planning/organizing patients' treatment (0.430)

**Factor 2 (5.1% variance; Cronbach's α = 0.7212): Making decisions**

Assessing patients' physical needs (0.635)
Making appropriate patient referrals (0.560)
Consulting with colleagues about care options (0.556)
Critically evaluating published research (0.516)
Analysing patient data (0.511)
Assisting patients in making informed choices (0.444)
Requesting laboratory investigations and results (0.441)
Appraising own and others' performance (0.416)

**Factor 3 (3.5% of the variance; Cronbach's α = 0.6899): Extending care provision**

Applying pharmacology to practice (0.675)
Assessing costs and outcomes of procedures (0.622)
Designing systems for patients' monitoring/observation (0.563)
Identifying areas worthy of investigation (0.556)
Liaising with other health care professionals (0.551)
Undertaking administrative duties (0.425)
Establishing a relationship with patients (0.305)

### Within-group comparisons

#### By work location: hospital versus community nurses (n = 300 and n = 224, respectively)

The training needs of the hospital-based nurses were compared with those of the community nurses to establish whether significant differences existed between them, which would merit the development of customized CPD programmes. A series of unrelated t-tests was performed on the difference scores (rating A – rating B), and 12 significant differences were found, with the hospital nurses having a significantly greater training need in each case. The results are presented in Table 6.

**Table 6:** Differences in training needs between hospital and community nurses (df = 522 in each case)

**Item**	**t**	**p**	**Mean score for community nurses**	**Mean score for hospital nurses**	**Implication**
Establishing a relationship with patients	-2.013	0.045	0.5759	0.7967	Hospital nurses have greater training needs
Applying pharmacology to practice	-2.362	0.019	0.4375	0.7067	Hospital nurses have greater training needs
Liaising with other health care professionals	-2.031	0.043	0.3750	0.6167	Hospital nurses have greater training needs
Consulting with colleagues about care options	-2.291	0.022	0.3393	0.5500	Hospital nurses have greater training needs
Recognizing and managing risk in clinical care	-2.720	0.007	0.4107	0.7000	Hospital nurses have greater training needs
Planning/organizing patients' treatment	-2.245	0.025	0.3839	0.5976	Hospital nurses have greater training needs
Assessing patients' psychological and social needs	-3.441	0.001	0.3937	0.7433	Hospital nurses have greater training needs
Prioritizing work according to patients' needs	-2.147	0.016	0.4152	0.6267	Hospital nurses have greater training needs
Undertaking health promotion/prevention activities	-2.713	0.007	0.4598	0.7467	Hospital nurses have greater training needs
Interpreting results from clinical investigations	-2.069	0.039	0.4688	0.6867	Hospital nurses have greater training needs
Actively assisting in change management activities	-2.9004	0.004	0.3482	0.6533	Hospital nurses have greater training needs
Planning patients' discharge	-2.268	0.024	0.2957	0.5100	Hospital nurses have greater training needs

These results suggest that hospital nurses have significantly greater training needs in almost a third of the tasks, thereby indicating that a customized set of CPD programmes would be relevant for each group of nurses, following a systematic review of their respective needs. The role of the community nurse is clearly reflected in this profile, because of their remit for health promotion and public health activities.

#### By grade of nurse

The training needs of each grade of nurse were analysed, first to establish whether differences existed between them and second, to identify the training needs of each grade. With regard to the first, the difference scores for the SPK, D3 and S1 were compared using a one-way ANOVA for unrelated designs. Only one significant difference emerged, which suggests that postbasic education packages would not need to be tailored for each grade of nurse.

With respect to the second aim, a series of related t-tests was performed on the difference scores (rating A – B) for each group. The results suggested that for both the D3 (n = 197) and the SPK nurses (n = 287), all tasks had a significant training need, in excess of p = 0.000 However, this method of ascertaining training needs simply identifies whether there is a difference between importance and performance ratings and therefore, it is possible to obtain a significant training need for a task which has a low importance rating. Inspection of the importance scores awarded to all tasks by the D3 and SPK nurses showed that 80% of the items were considered to have importance ratings of >6.00, with the remaining 20% having importance scores between 5.5 and 5.9. Thus all tasks were considered to be highly relevant to the work of both the D3 and SPK grade.

However, for the S1 nurses (n = 40), five tasks recorded no training need, while the significance of training need for the other 35 tasks varied between p < 0.00001 – p < 0.05. These results are classified by order of significance in Table 7.

**Table 7:** Training needs for S1 nurses, in priority order of significance

**Significant at p < 0.00001**

Establishing a relationship with patients
Designing systems for patient monitoring/observation
Assisting patients in making informed choices
Assessing patients' physical needs
Recognizing and managing risk in clinical care
Assessing patients' psychological and social needs
Undertaking health promotion/prevention activities
Developing joint working relationships with others
Interpreting results from clinical investigations
Instructing/training students/junior staff
Undertaking clinical examination of patients

**Significant at p < 0.001**

Making appropriate patient referrals
Planning/organizing patients' treatments
Working as a member of a team
Developing shared mission of clinical goals
Using technical equipment
Writing clinical/shift/other reports
Collecting own clinical/patient/surveillance data
Actively assisting in change management activities
Interpreting own patient data

**Significant at p < 0.01**

Identifying areas worthy of investigation in own practice
Liaising with other health care professionals
Analysing patient data
Appraising own and others' performance
Critically evaluating published research
Prioritizing work according to patients' needs
Introducing new ideas into own clinical work
Inputting data into written or computerized records
Planning patients' discharge
Locating/accessing relevant equipment for clinical work

**Significant at p < 0.05**

Applying pharmacology to practice
Assessing costs and outcomes of procedures
Consulting with colleagues about care options
Making decisions about patients' clinical problems
Getting on with colleagues

As with the other commentary about the absolute importance of each task, relative to the associated training need, 31 tasks were accorded importance rating in excess of 6.0, seven had importance ratings between 5.43 and 5.98, and two (undertaking administrative duties and budget planning activities) were scored as being of only moderate importance. These results, then, demonstrate that the training needs of the S1 nurses can be differentiated according to their significance, which would afford the limited training budgets to be targeted at the areas of greatest importance.

#### Training needs by province

The training needs of nurses from each of the five provinces were compared using a series of one-way ANOVAS, with post hoc Tukey tests in the case of significant results. Twenty three differences were obtained, with East Kalimantan and North Sulawesi (the least developed of the five provinces) typically recording greater training needs than Jakarta, North Sumatra and DKI. As only five provinces (out of the 34 at the time of writing) were sampled, few conclusions can be drawn about the pattern of training needs by province across the whole of Indonesia. However, what these results do underline is the huge difference between provinces and the need for systematic training needs surveys to be conducted prior to mounting any CPD provision.

### Impact of having a job description

In order to identify whether the provision of a formal job description affected nurses' perceptions of their occupational role, the importance ratings for each of the 40 tasks recorded by those nurses who had a job description (n = 276) were compared with those recorded by the nurses without a job description (n = 248), using a series of unrelated t-tests. Only five differences emerged, indicating that the value of a job description (in its current format) in defining boundaries, function and occupational framework is limited.

Similarly, those nurses with and without a job description were compared on their reported training needs, to ascertain whether a formal job specification enabled greater insight and recognition of where skill deficits lay. Using a series of unrelated t-tests, only two differences were found, thereby suggesting that possession of a job description made little impact on respondents' understanding of their own development needs relative to their job specification.

### Impact of having an individual performance assessment (IPA)

To establish whether feedback about individual performance via the annual Government IPA affected how the nurses perceived their own performance evaluation, those nurses who had had an IPA (n = 411) were compared with those who had not (n = 113), on their self-reported performance assessments (rating B). Using a series of unrelated t-tests, only four differences were found (10% of tasks), thus indicating that the IPA system does not affect self-perceived performance evaluations. Using the same protocol for analysis, the training needs of each group were also compared and again only four differences emerged. Together these results emphasize the lack of impact that feedback through the current IPA system has on nurses' perceptions of their own performance and development needs.

## Discussion

The results will be discussed in the order in which they were presented in the preceding section.

### Occupational profile of nurses: whole sample

All tasks in the questionnaire were considered to be important, with 34 (85%) considered to be extremely important. Because of these ratings, it is neither possible nor relevant to prioritize the tasks according to perceived salience, since it is self-evident that all items are deemed to be crucial to the sample's jobs. This may reflect the pressure on the nursing workforce to undertake all activities that arise, irrespective of formal role definition, as a direct result of the shortfall in available personnel and resources.

To ascertain whether these tasks clustered together in coherent groupings that could be used as a basis for curriculum development in basic nurse training, an exploratory factor analysis was undertaken. Three factors emerged, which together accounted for almost half the variance. The first factor, provisionally entitled "Clinical care and service management" comprised Factor 2 (reflective practice) from the original factor analysis [8] and much of factors 3 (decision making) and 4 (technical and administrative procedures) [8]. Factor 1 in this paper has two clear components – the development of reflective practice and technical administration. These components could form two unified modules in the basic curriculum of the nurse-training programme. The presence of the reflective practice items loading on this factor resonates with the recently introduced national policy for a clinical performance development and management system for nurses and midwives in Indonesia [13], which encourages nurses to become more reflective in their clinical work, through regular audit, peer review and case discussions [14].

The second factor, provisionally entitled "Managing change and the application of knowledge", contained items that indicated high-level clinical work and mirrored the first factor emerging from the original factor analysis [8]. The third factor to emerge, labelled "Case management and networking" maps closely with factors 5 and 6 from the initial analysis [8]. This factor highlights the importance of liaising with other health care groups in order to manage patient care effectively, through a system of professional contacts. Together these factors present a logical framework for developing a basic nurse training programme, using a modular structure for delivery and assessment that is founded on the contents of each factor. Moreover, all factors were internally reliable, confirming the psychometric merit of the instrument.

These items, because of the criticality accorded to them by the respondents, could be used to formalize generic job descriptions in a more systematic and informed way. Implementation of clear role boundaries might go some way towards ensuring that nursing care does not exceed the limits of practitioner competence, thereby managing risk; it would also facilitate the annual performance assessment, by providing clear benchmark statements against which to measure performance and through which future CPD needs can be identified and met.

### Occupational profile of nurses: subgroup comparisons

#### By province

However, the diversity of health care problems and the context in which they occur and are managed means that a generic job description may be only a starting point for many nurses, with local issues determining some aspects of the job specification. The environmental obstacles mean that some geographical areas are virtually inaccessible, have very limited equipment and few nurses, inevitably creating profound differences in how nursing care can be delivered. It would be surprising, then, if the items contained in the questionnaire had the same relevance for all the respondents.

Therefore, to identify whether there were any significant differences in perceived occupational role within different locations, nurses from five provinces were sampled and their responses to criticality of tasks were compared. Thirteen significant differences in reported criticality were found (32.5% of tasks), suggesting considerable variation in job roles across these provinces. No coherent pattern in the differences appeared, either in the nature of the tasks or how the provinces construed them. However, that almost a third of the occupational role varied by province suggests that nursing function is heavily influenced by local factors and that it may be valuable to draw up job descriptions that reflect this diversity.

As only a small sample of nurses from just five provinces participated in the current survey, the results cannot be generalized across the whole of Indonesia. However, there may be merit in using this questionnaire approach to delineate the role boundaries across the country, such that locally relevant training packages, job descriptions and IPA frameworks can be developed.

#### By hospital and community nurses

Similar comparisons were undertaken for hospital and community nurses. Eight significant differences emerged, thereby suggesting an 80% overlap in job function between these two groups of nurses. No clear pattern was identified in those tasks where differences were found. The accord between the two sets of rating may be not be sufficient to justify the cost implications incurred in devising basic training courses, customized to meet the needs of these two sections of the workforce. On the other hand, it may be exactly the fact that there is currently one only preparation for hospital and community nurses that has led to the limited differences observed between their roles. However, it might be feasible to mount some short-course CPD provision that takes account of the distinctions in job function, and to modify job descriptions and IPAs with this in mind.

#### By grade of nurse

The results obtained from the comparisons of criticality rating awarded by the three grades of nurse (SPK, D3 and S1) also showed significant overlap in core job function (33 of the 40 tasks), without any coherent pattern to the differences being apparent. While the distinctions between the grades are few, that some existed at all may be a reflection of the different ways in which nurses are prepared for their roles as well as the career pathway each grade is most likely to follow, with the resultant differences in the manner in which the duties are discharged [2,14].

It is worth pointing out that the qualitative data obtained during the focus group sessions, conducted during the preparatory stages of the study [8], revealed appropriate differences in how the tasks were conceptualized by the various grades. For example, for all nurses the item "applying pharmacology to practice" meant handing out medicines prescribed by a doctor. However, the S1 nurses also understood this to mean knowledge about the nature of the drug, assessing the patient before administering the dosage and anticipating possible side effects. This enhanced understanding of the task was a clear reflection of the higher levels of background education experienced by the S1 nurses. However, to take account of these variations in role conceptualization, specific job descriptions might be relevant, with all the regulation of function, preparation and practice this would afford.

### Training needs

#### Whole sample

To ascertain the training needs for the whole sample, the performance ratings were deducted from the importance ratings [11]. These comparisons demonstrated that the nurses sampled had highly significant training needs on all 40 items. As significant training needs can be produced for tasks that have only limited importance (i.e. poor performance of a task awarded only moderate salience), it is clearly essential to consider the absolute value of the importance ratings, in order to establish a priority listing of development needs, which can be targeted for CPD provision.

For the current sample, 34 of the tasks (85%) were considered to be highly crucial to the respondents' jobs, with importance ratings in excess of 6.00. The remaining six tasks achieved importance ratings of between 5.5 and 5.89, suggesting that while not of primary relevance, these tasks were also deemed to be crucial. The activities in this category included assessing costs and outcomes of procedures, making appropriate patient referrals, critically evaluating published research, undertaking budget planning activities, actively assisting in change management activities, and planning patients' discharge. These findings indicate that management of systems, budgets and patient pathways may not be a core role of the nurse in Indonesia.

However, to determine whether the nurses' training needs fell into clusters of related activities that might inform the module and course content of postbasic training, an exploratory factor analysis was performed, using a Varimax rotation. This process yielded three factors, which have been provisionally labelled "Nursing care and leadership" (factor 1), "Making decisions" (factor 2) and "Extending care provision" (factor 3). These factors, detailed in Table 5, do not reflect the factor structure emerging from the factor analysis of the criticality ratings, which suggests that only selected aspects of each of these factors requires further CPD. In other words, the construction of CPD modules should not be a simple reworking or updating of the modules identified for basic training, but should reflect the actual skill deficits reported by the sample.

At a broader level, these findings indicate, first, that the methodology used may be a sensitive measure of actual development needs, and second, that effective CPD provision can be customized to meet the needs of potential participants in this way. The wider implications of this are that limited educational budgets and resources can be efficiently deployed to meet the actual shortfall in skill levels. This is in direct contrast to the standard model of CPD provision in many countries, where CPD provision represents the views and assumptions of strategic planners and managers within the health service and which, in developing countries, may be driven by the idiosyncrasies and agendas of donor loans and grants rather than the real development needs of the health care professionals themselves.

### Training needs: subgroup comparisons

#### By work location: hospital versus community

Because of the diversity of work locations that result from the structure of the Indonesian health service, the particular demands of the health issues that arise and the huge geographical variations across the country, it would be reasonable to predict that how nurses discharge their duties would be heavily determined by the context in which they operate.

One key contextual difference relates to hospital and community staff, many of the latter working in inaccessible and hostile terrain, with few resources and limited staff and equipment. Consequently, the development needs of these nurses may differ significantly from hospital-based nurses, which in turn might merit customized CPD provision for each group.

To test this assumption, the training needs of the 300 hospital nurses were compared with those of the 224 community nurses (see Table 6). These revealed 12 significant results, with the training needs of the hospital nurses being greater in each case. Many of these are predictable, in that they relate to tasks and situations that might prevail more in hospitals than in the community, such as consulting with colleagues, liaising with other health care professionals, interpreting results from clinical investigations, etc.

The extent of the training needs difference between the two groups (almost a third of the nursing role) could imply the need for a more intensive programme of CPD with the hospital nurses, as a matter of priority, thereby optimizing the use of the available education budget. However, as community nurses (especially those in remote areas) have very little opportunity for CPD, they may be unaware of their own development needs or that they need to enhance their own performance. In any event, the results confirm the importance of undertaking a systematic review of need before mounting any CPD provision.

#### By grade of nurse

Because of the recent radical changes to basic nurse education in Indonesia (i.e. the cessation of SPK training and the increase in D3 and S1 educational provision), it might be reasonable to suppose that the levels of competence attaching to each grade and arising out of distinctive basic nurse education preparation might engender different development needs for each grade. Comparisons of training needs across the three grades, however, yielded only one difference between them ("establishing a relationship with patients"). These results indicate that all grades of nurses have very similar development needs and that separate, customized CPD provision would be unnecessary. However, it should be noted that, as with the occupational profile results, the detailed interpretation of each item made by different grades of nurse during the preparatory focus group sessions revealed subtle variations in levels of understanding. These differences should be considered when developing CPD programmes for each grade.

With regard to the actual training requirements of each grade, the D3 and SPK nurses all recorded highly significant training needs for all 40 tasks (p < 0.0001), with 80% of the tasks also being considered to be very salient to the respondents' roles, and the remaining 20% of tasks receiving importance ratings of between 5.55 and 5.9. The challenge for educational providers, then, would be to generate a coherent group of CPD modules that would mirror the clusters of training needs identified, and that could be provided to both grades of nurse, perhaps in a shared learning environment. For the more highly educated S1 nurses, five tasks did not require further training, while the remainder fell into four different strata of urgency, according to the associated level of significance (see Table 7). With limitations on available training budgets, it would seem expedient to focus primarily on those tasks falling into the categories of greatest need, especially establishing a relationship with patients, designing systems for patient monitoring/observation, etc., which are essential components of clinical practice.

In taking account of the need to examine the absolute importance ratings for each task, as well as the significance of the training need, the study found that only two tasks fell below a mean importance rating of 5.43. These were "undertaking administrative duties" and "budget planning activities", and may reflect the fact that S1 nurses move quickly into nurse education on completion of their own training, which would limit the salience of such tasks. Nevertheless, as teachers, they would have to be able to teach their students about such topics.

#### Training needs by province

Comparisons of the training needs of nurses from each of the five provinces sampled revealed 23 differences between these localities. No distinct pattern emerged, although the needs of nurses in East Kalimantan and North Sulawesi were typically greater than elsewhere. Because only five provinces out of the total of 34 were represented in this study, it is impossible to identify trends or definitive areas of training need that would apply across the country. However, the vast number of differences between the regions studied strongly suggest that nurses' development needs are determined to a large extent by where they work, thus there is a need to focus on CPD packages that are pertinent to each province. In this way, customized development can be offered which in turn should positively affect the health and clinical care of patients.

### Impact of having a job description

The flexibility with which nurses discharge their duties is in part a function of the situations in which they work, the needs of patients and the availability of other health care professionals to share the provision of care. These factors notwithstanding, the lack of a formalized definition of the nursing role has contributed to a situation whereby nurses may potentially exceed their boundaries of competence and authority in order to deliver care. This may have a deleterious impact on both patient well-being and professional credibility.

The presence or absence of a job description could conceivably affect how the nurse construes the limits of the role; this might be reflected in his/her understanding of the criticality of the tasks included in the questionnaire, and it might be reasonable to suppose that those nurses with a job description have a different understanding of the parameters of their role. However, comparisons of the importance ratings awarded to each of the 40 activities by nurses with job descriptions, and those without, revealed only five significant differences. This implies that possessing the kind of job specification available at the time of the survey has little impact on how nurses conceptualize their occupational profile.

One explanation for this could be the lack of specificity of the typical job description, which at the time of the survey were generally inadequate for role clarification. This therefore created fuzzy boundaries to the nurses' roles and thus offered the potential for a wide range of interpretations to be placed on both the nature and level of the nursing tasks undertaken.

Similarly, the possession of a job description made little difference to how the respondents reported their development needs. This is hardly surprising, in that the absence of a clear statement of role responsibilities makes it impossible to benchmark how current performance relates to these, and thus makes any assessment of skill deficit and training needs difficult to identify. The earlier synopsis of the nursing role could be used as a framework for tightening the specification and thus for regulating the way in which the nursing role is conceptualized, developed and delivered.

### Impact of having an IPA

The lack of an agreed job specification also makes it difficult both for an external agent, such as a manager, to assess a nurse's performance, as well as for the nurse him/herself, because there are no criteria against which to monitor it. This undermines the government's attempts to introduce a mandatory IPA system for all public sector employees, as well as making the workforce's CPD needs difficult to capture.

It might be expected that those nurses who had experienced a recent IPA would have a different understanding of their current work performance and their future development needs, compared with those nurses who had not been in receipt of this. However, comparison of these two groups on current performance ratings (rating B) and training needs identified only four significant differences in each set of comparisons, thus suggesting a limited impact of the mandatory IPA. As the IPA system is not fully in force, with the system ranging from nonexistent through casual to formal review, it is conceivable that very few nurses have a clear understanding of the levels to which they should be operating. The information provided by this survey might help to inform both the process and the competences that require external assessment.

## Conclusion

The results of this survey demonstrate that there is little difference in how different grades of nurses perceive the nature of their role; in turn, this may reflect that in practice, nurses have to discharge whatever care is needed, irrespective of limits of competence. There is, then, clear need for specific job descriptions and competence levels by grade of nurse. This study has also shown the distinction in training needs by type of nurse and locality of work, which serves to demonstrate the importance of collecting systematic data prior to developing postbasic training packages.

While the results of this study are only indicative, partly because of sampling issues and partly because of the fluctuating nature of the Indonesian health and economic system, they do make a general point: that the instrument used here has the capacity to elicit reasonably objective data in a systematic way, which would enable education providers and service managers to develop the nursing profession both pre- and post-registration, in a way that meets local needs and issues.

The findings from this survey should go part-way to informing the process of defining roles and competences, as part of the wider agenda of developing health care professionals and service provision. Indeed, some preliminary initiatives emerging from this work have already been implemented.

The results of the study were disseminated to the Ministry of Health and Professional Associations, on the basis of which a new model of management and quality control for nurses was developed, with the support of WHO. The model is called "Clinical Performance Development and Management System for Nurse and Midwives", and is built on the concept that good pre-service theoretical and clinical education is an essential foundation on which to build structures and processes for developing high-quality health care. These structures include needs-based job descriptions, defined clinical standards and guidelines, continuing education, performance indicators and clinical management skills [14]. The processes include leadership and support for high quality care, monitoring the performance indicators, feedback of results, educational development and training to reach the required standards and regular reflective case discussions as part of continuing professional development [14].

The model has been introduced in hospitals and communities throughout nine provinces [15]; the programme began in 2001, by 2004 it had become national policy supported by all the departments in the Ministry of Health and in 2005, its implementation across the whole of Indonesia was supported by ministerial decree. In the intervening years, the system was developed and improved and was introduced into a third of all provinces. The early indications are that the scheme has improved the focus on nursing standards and there has been a greater understanding of a positive impact of a defined boundary of activity through detailed job descriptions. The preliminary success of the programme [14] encouraged the Ministry of Health, in partnership with WHO, to seek the resources necessary for further expansion, with a very encouraging response from both the Government of Indonesia and donors.

## Competing interests

The author(s) declare that they have no competing interests.

## Authors' contributions

DH designed and managed the survey and organized its data collection and preliminary analysis, assisted by AH and YK; CH did a secondary data analysis and wrote the article. All authors read and approved the final article.
